# Neuroethical considerations and attitudes about neurostimulation as a fatigue countermeasure among emergency responders

**DOI:** 10.3389/fnrgo.2024.1491941

**Published:** 2024-12-18

**Authors:** Laura Y. Cabrera, Alejandro Munoz, Ranjana K. Mehta

**Affiliations:** ^1^Department of Engineering Science and Mechanics, Center for Neural Engineering, and Rock Ethics Institute, The Pennsylvania State University, University Park, PA, United States; ^2^Cabrera Lab, The Pennsylvania State University, University Park, PA, United States; ^3^Department of Industrial and Systems Engineering, University of Wisconsin Madison, Madison, WI, United States

**Keywords:** neuromodulation, fatigue, neuroethics, perspectives, first respondents

## Abstract

**Introduction:**

First responders play a pivotal role in ensuring the wellbeing of individuals during critical situations. The demanding nature of their work exposes them to prolonged shifts and unpredictable situations, leading to elevated fatigue levels. Modern countermeasures to fatigue do not provide the best results. This study evaluates the acceptance and ethical considerations of a novel fatigue countermeasure using transcranial Direct Current Stimulation (tDCS) for fire and emergency medical services (EMS) personnel.

**Methods:**

To better understand first responders' perceptions and ethical concerns about this novel fatigue countermeasure in their work, we conducted semi-structured interviews with first responders (*N* = 20). Interviews were transcribed into text and analyzed using qualitative content analysis.

**Results:**

Over half of responders (59%) were interested, but over a third had a cautionary stand. Half of the participants seemed to have positive views regarding acceptability; a few were more cautionary or hesitant. A main area of consideration was user control (75%), with the majority wanting to retain some control over when or whether to accept the stimulation. Just above half of the participants (64%) mentioned privacy concerns. Another relevant consideration, raised by 50% of participants, was safety and the potential impact of stimulation (e.g., side effects, long-term effects). Overall, participants thought they needed to understand the system better and agreed that more education and training would be required to make people more willing to use it.

**Discussion:**

Our exploration into combating fatigue among first responders through tDCS has revealed promising initial reactions from the responder community. Findings from this study lay the groundwork for a promising solution, while still in a nascent design stage, to improve the effectiveness and resilience of first responders in fatiguing shifts and critical situations.

## 1 Introduction

There has been an expansion of neurotechnologies beyond medical applications to fields like wellness (Kreitmair, 2019; Hendriks et al., 2019; Paek et al., 2020), military (Ienca et al., 2018; Sattler et al., 2022), entertainment (Paek et al., 2020), education (Williamson, 2019; Privitera and Du, 2022), and workplace (Muhl and Andorno, 2023; Midha et al., 2022). In high-risk work domains, such as emergency responders (ER), the ability to track and manage fatigue is critical for enhancing worker safety and performance. Fatigue—the subjective, unpleasant symptom, which incorporates total body feelings ranging from tiredness to exhaustion—creates an unrelenting overall condition that interferes with an individual's ability to function to their normal capacity (Ream and Richardson, 1996). Fatigue reflects the body's strategy in resource management, and as such, it has several physiological roles, including energy conservation and homeostasis maintenance. Fatigue impacts performance via gradual deficits in working memory (Taverniers et al., 2010), perceptions of exertion (Mehta and Agnew, 2015), and attention (Hancock and Warm, 2003), all of which impact situation awareness (Cak et al., 2020), and decision-making, and judgment (Paton and Flin, 1999). Fatigue among ER workers, including fire service and emergency medical services (EMS), has been associated with 3-fold increases in injury and depression risks and a four-fold increase in health and safety-compromising behaviors (Patterson et al., 2012; Rusiecki et al., 2014). ER operations require extended shifts, sustained periods of wakefulness, and prolonged periods of decision-making in highly demanding, unpredictable environments (Aisbett and Nichols, 2007; Mehta et al., 2020; Peres et al., 2023a). These preconditions make both physical and mental fatigue among ER professionals a common experience, impacting their operational performance, as well as their psychological readiness, grit, and resilience (Kauffman et al., 2022).

Numerous attempts have been made to reduce the debilitating impacts of responder fatigue (Peres et al., 2023b), such as regulating work schedules (Lee, 2011), implementing sleep intervention programs (Jang et al., 2020; Sullivan et al., 2017), and training responders to manage their sleep and lifestyle behaviors (Barger et al., 2018). These countermeasures are fundamentally limited in their timeliness and are repeatedly de-prioritized due to operational and organizational constraints, particularly in times of local and national emergencies. ER workers often end up responding to fatigue by taking stimulants, which come with substantial side effects (Manchester et al., 2017), are difficult to ingest while in the field, and do not address the diverse effects of fatigue (Eastlake et al., 2015). There is a critical unmet need to develop transformative and responsible approaches to proactively monitoring, predicting, and managing fatigue that protects responder health and safety, as well as the safety of the communities they serve.

Non-invasive brain stimulation (NIBS) presents a potential alternative to preserving human performance under extreme fatigue and has been shown to have comparative advantages over caffeine in managing the deleterious effects of fatigue in a timely fashion (McIntire et al., 2017, 2014). Transcranial direct current stimulation (tDCS) is a NIBS technique that aims to alter brain function and is inexpensive, safe, temporary, reversible, and is an effective way of augmenting a variety of cognitive abilities (Brunoni et al., 2012; Reato et al., 2019). tDCS has shown to be an inexpensive, safe, temporary, reversible, and effective way of preserving/augmenting cognitive functions in military, consumer, and clinical settings (Peltier et al., 2019; Fregni et al., 2005, 2020). Many studies have found that stimulation of different brain regions with tDCS can enhance the performance of basic cognitive tasks that recruit the corresponding brain regions (Fregni et al., 2020). Studies have shown the strong potential of tDCS to improve operator situation awareness and performance on critical first-response tasks, such as surveillance and threat detection (Clark et al., 2012; Falcone et al., 2012; Parasuraman and Galster, 2013). Similarly, tDCS shows beneficial effects on vigilance (Karthikeyan and Mehta, 2020) and working memory (Karthikeyan et al., 2021), with some studies reporting cognitive improvements during extended periods of sleep-deprivation-induced fatigue (McIntire et al., 2017, 2014). Other studies have focused on the role of tDCS in mental fatigue (Nikooharf et al., 2022) and fatigue relief related to disease conditions such as multiple sclerosis (Chalah et al., 2015; Tecchio et al., 2022), stroke (Doncker and Ondobaka, 2021), and polio (Acler et al., 2013).

Neuroethics, a field that focuses on ethical and societal implications of advances in neuroscience and neurotechnology (Farah, 2012), has examined ethical considerations raised by these advances and neurotechnologies in clinical and when used for enhancement purposes (Hendriks et al., 2019; Eaton and Illes, 2007; Ienca and Andorno, 2020; Zuk et al., 2018). More recently, it has started to delve into questions of neurotechnology in other domains; an example is the work of the IEEE BRAIN Neuroethics Subcommittee and the proposed neuroethics framework that looks at nine different domains of applications, with workplace being one of them. The ethical considerations and implications of tDCS use have been studied in the clinical and military domains (Lavazza, 2019; Davis and Smith, 2019; Feltman et al., 2020; Day et al., 2022; Auvichayapat and Auvichayapat, 2022). Yet, not much research has explored ethical considerations and barriers to the use of tDCS in the workplace. Prior research shows that successful adoption of new interventions depends on factors such as organizational or domain culture, social interaction, and workers' perceptions of functional usefulness and ease of use. Thus, this is important as it can impact the usability of the system. To address this gap, this study explores attitudes and ethical considerations about a tDCS-based fatigue countermeasure aimed at mitigating the debilitating impacts of fatigue on ER workers.

## 2 Methods

We conducted semi-structured interviews with 20 US-based emergency responders, either actively engaged or retired from the field of duty or involved in ER training during 2021.

### 2.1 Recruitment and participants

We contacted potential participants by emailing flyers through a local ER training facility to nearby fire and EMS agencies with an invitation to participate in the study.

We enrolled them into the study if they were at least 18 years old, were either current or retired ER personnel or otherwise involved in ER training and spoke comfortably English. When scheduling each interview, we sent participants a letter that described the study and stated that proceeding with the interview indicated their voluntary agreement to participate. The study was approved by the Texas A&M University Institutional Review Board (Exempt determination IRB2021-1342M). There was no compensation for participation in the study.

Participants were informed before the start of the interview about their right to decline to answer any questions or to decide to stop or withdraw their involvement at any point during the interview. A team member conducted each interview with participants in person or by video teleconference (Zoom). All our participants were male, with different positions (firefighter/paramedics, captains, assistant EMS managers, agency instructors, chiefs, education supervisors, program managers, president of a relevant organization, and program director) at various organizations, and had a wide range of experience (the mean number of years of experience in the field was 17.8 years).

### 2.2 Procedures

We conducted semi-structured interviews to ensure consistency, as well as to facilitate the exploration of unanticipated issues and in-depth understanding while covering a core set of topics (Miles et al., 2013). We used Zoom transcription and revised the transcription for each interview. Each interview lasted between 30 and 40 min. We first asked participants some general questions about their roles and responsibilities. We asked them about the challenges they face in their roles, in particular, their thoughts about fatigue and fatigue management methods. Then, we introduced the technology proposition and captured their initial reactions, followed by questions about their concerns, opportunities, and barriers. We ended up discussing other concerns or views they might have related to the proposed solution.

### 2.3 Data analysis

We analyzed the text in the interview transcripts using qualitative content analysis methods and a deliberative approach (Hsieh and Shannon, 2005; Sandelowski, 2000). We used Excel to help us organize the data for analysis. We analyzed the first two transcribed interviews and created a draft codebook. All themes were reviewed and refined by two coders [LC, AM]. We based several codes on core aspects of the questions asked during the interviews. We analyzed another two transcripts to see if the codebook needed to be adjusted. We used the developed codebook to code the rest of the transcripts. Team meetings provided opportunities to reach a consensus on coding discrepancies. As part of the analysis, the perspective and positionality of researchers have been acknowledged (Elliott et al., 1999). All researchers are based in the US, but two were not born in the US. In our Results section below, we present relevant quotes to highlight the findings with non-content words and expressions removed for readability.

## 3 Results

We categorize results into three main groups: (1) fatigue and countermeasure fatigue views, (2) tDCS attitudes and concerns, and (3) views on acceptability. For each of these main categories, we identified several themes.

### 3.1 Fatigue and views on fatigue countermeasures

The first category captured views relating to fatigue and fatigue countermeasures and includes the subthemes (1) fatigue impact, (2) fatigue countermeasures, and (3) views on ideal solutions to counter fatigue.

Only half of the participants shared their views on fatigue, with several of them (*n* = 6/10) mentioning that fatigue has both a mental and physical dimension, associating fatigue with tiredness from sleep deprivation and working long shifts.

“I think about it on two different levels. One is just sort of mental fatigue […], And then I also think about physical fatigue, especially, many responders are on duty for long hours.” (Participant 07)

More than half of the total participants (*n* = 13/20) mentioned the impact of fatigue in their work, including putting their safety and that of others on the line, as it increases the chances for more accidents, more mistakes, and not being able to think clearly.

“You know, no one wants to be on shifts when they haven't slept the night before because not only is it dangerous to the patient, but it's also dangerous for you because you can prove yourselves and unsafe situations.” (Participant 04)

Participants shared a few fatigue countermeasures they use to keep fatigue at bay, as well as some views they have about them. Almost half (*n* = 8/20) reported coffee as their number one fatigue countermeasure, or even if not coffee drinkers themselves, they acknowledged it as a clear trend among their peers. Exercise was the second most mentioned fatigue countermeasure, followed by taking naps, as well as eating well, and staying hydrated. A few (*n* = 3/20) mentioned drinking energy drinks, and one mentioned using Adderall. Some mentioned a combination of different countermeasures.

Participants also discussed their ideal solutions to fight fatigue (*n* = 9/20). Most of these participants (*n* = 5/9) mentioned shift length restrictions, dividing work hours, having more people on shifts, and having more breaks. One person mentioned having more personnel, and another had an extra ambulance.

“[R]educing our hours, putting in more breaks, investing in, having more people on shifts, and alternating shifts, rather than trying to reduce our fatigue.” (Participant 04)

### 3.2 tDCS attitudes and concerns

This category captured attitudes and concerns relating to the use of tDCS and included the subthemes of user control, privacy, safety concerns, misuse, and mistrust.

Most participants who voiced a reaction to tDCS mentioned being interested (*n* = 10/20), however, a sizable number (*n* = 7/20), including cases of people who mentioned that it sounded interesting, also reacted with caution. Only four participants had a very positive reaction, saying this was a fantastic idea or that they loved the idea.

“So initially, whenever you talk about brain stimulation, I'm thinking about shock therapy […] So I can't say that my first initial thought on that is positive. … but I'm open.” (Participant 03)

“I'd probably be hesitant, but by looking at research, I'd be willing to try it.” (Participant 05)

More than half of participants (*n* = 14/20) mentioned user control as a concern, how the device would be controlled when in use, with the question of there being an automatic or manual option. There were diverse views on the specifics, with some wanting the system to only monitor fatigue and not stimulate, only one participant was upfront about wanting the stimulation to be automatic, and the rest talked about their preference for keeping control, at least until they were comfortable or trusted the system.

“It would have to be in a sort of on-demand kind of thing, where I initiate the stimulation because if I'm in the middle of starting an IV, and then all of a sudden, this thing shocks me, it could be very distracting” (Participant 03)

“[O]n/off button for the device would seem to be a good feature it would allow me to feel more in control of the device, if there's no on-off feature, stimulating my brain, it kind of sounds pretty Orwellian. I wouldn't want something controlling my brain without my permission.” (Participant 04)

“[When] people have adopted the technology and kind of accepted it, and it had become kind of an industry standard, I think automation is a great thing at that point.” (Participant 09)

Just over half of the participants (*n* = 11/20) mentioned concerns about privacy, including considerations about sharing data recorded by the device, who has access to the data, where the data goes, and how it might be used. Some participants raised considerations related to liability, for example, if the recorded data was taken out of context negatively by the employer. Others reinforce the need to make sure the systems are secure, considering the type of information being collected.

“What could this data reveal about you that maybe was unintentional? […] are you going to end up revealing something that you weren't aware […] So I think that the privacy concern would be there.” (Participant 07)

Half (*n* = 10/20) mentioned safety considerations and the potential impact the stimulation would have on a person, with all except for two focusing on the safety risks, including long-term effects, unintended side-effects, the possibility that it would affect your circadian rhythms, and safety considerations of pushing our natural limits. Others wanted to know if the stimulation was going to continue to have the same effects throughout time or if it would either become less effective or bring about new effects.

“You don't want, I think, put people at risk if you're using a device for longer than it needs to be utilized” (Participant 11)

About a third of our participants (*n* = 6/20) raised concerns about the potential for misuse, either by the end-user or by the employer. Concerns were mentioned that people could essentially use it to their advantage by abusing the stimulation. A related concern mentioned by a similar number of participants (*n* = 6/20) was related to pushing someone's natural limits, though one participant mentioned this is not a concern because we humans do this all the time. A few also mentioned the possibility that employers will just keep pushing their employees' limits by using this.

“[C]ould be used incorrectly, either by the end-user or the […] employer, … now we can do this, and I can push this person this much further. So now I don't have to have this other XY crew that costs this much money.” (Participant 06)

“It depends on who's using this…if someone's using it to gain a competitive advantage…that's not a good thing” (Participant 19)

A final and related concern to several of the ones mentioned above is related to trust (*n* = 3/20), including not trusting the system or being more comfortable with control of the system once you trust it.

“I do have a little bit of an issue with an external device controlling the way that I think” (Participant 20)

### 3.3 Views on acceptability

This final category captured views relating to views on acceptability and included the subthemes of general views on acceptability, facilitators, and barriers.

While overall, the system was acceptable (*n* = 10/20), four participants were more cautionary, and three explicitly mentioned that there would be hesitation (both from their perspective or others in the field). A quarter (*n* = 5/20) of participants mentioned that this system makes sense and even brings promise to the EMS community, as this type of public safety jobs need to keep on top of things that can help those working these types of jobs, in particular, tracking fatigue can help people to mitigate harms to the community they aim to serve and to help them do their jobs better. Another third of the participants had some acceptance but were cautionary and perceived the countermeasure as akin to science fiction. They needed to understand better what the system does, its side effects, and whether the benefits outweigh the risks. Others reflected that not everyone will be on board, that it is likely that people will be hesitant until it becomes more widely used, and that education and training should occur to make people more likely to want to use it, but not make this mandatory for those working this type of positions.

“I think [acceptance] would be slow at the beginning. But if it was proven to be effective […] it'd be a tool that they would probably use and embrace” (Participant 06)

“We're actively trying to make our work shift shorter and to lessen the work, but at the same time, we know that there are going to be times when they're going to become fatigued, and we need a safety net that can prevent them from getting into a dangerous area” (Participant 10)

### 3.4 Facilitators

Participants mentioned several considerations that will make acceptance easier by first responder workers. A key factor mentioned by several participants (*n* = 8/20) was having the system be convenient (e.g., that enables them to move easily and comfortably). Other considerations included mentioned that it is easy to use (e.g., as a wearable, watch or integrated into their uniform), lightweight and compact, that they could connect to their phone as an app enabling self-monitoring or interaction with the device, and the need to be resistant to heat, water and/or sweat depending on the type of job. Maintenance, durability, battery life, and the importance of having multiple layers of security and encryption were also mentioned by participants as important factors.

“[Something that] it's easy, and it's not invasive, and it can integrate into existing gear, or it can integrate into a hat or something like that and not look dumb, it's a big thing.” (Participant 12)

### 3.5 Barriers

There were also several barriers noted by participants. For example, several mentioned that if the system was too weird looking or made them look silly (*n* = 6), it could cause mistrust among those they are trying to serve and become a barrier to their work. Three participants were very explicit that a headband (or something placed on the head or neck) would not be welcomed by them. Finally, the cost to the departments running this type of job can be a barrier to uptake, as well as some of these jobs having more traditional mindsets that make them hesitant to change.

“I don't think anyone wants to look weird in public. Right? We're in the public eye all the time. So if you're wearing electrodes on your head, people are like, no, no, we don't want your help.” (Participant 2)

“I think the biggest barrier is people understanding how it's going to benefit them.” (Participant 16)

## 4 Discussion

Neurotechnology in the workplace is a growing area, and while literature is growing in producing guidelines to mitigate the negative implications (OECD, 2019; Goering et al., 2021; UNESCO, 2023), very few are focused on the workplace application or workers' views. Our results suggest that there are important reasons to consider fatigue countermeasures. However, several participants are divided on their acceptability of technological-based ones, raising concerns that such measures can potentially push human capacities to unhealthy limits or that the data recorded might not be used necessarily for their benefit. The main ethical concerns raised were regarding privacy and user control, which align with recurring concerns outlined by various authors regarding stimulating neurotechnologies (Lavazza, 2019; Davis and Smith, 2019; Feltman et al., 2020; Day et al., 2022; Auvichayapat and Auvichayapat, 2022; Farah et al., 2014).

### 4.1 The realities of fatigue countermeasures strategies

Current fatigue mitigation strategies in ER are fraught with challenges, rendering them inadequate for the responders. For example, our study respondents reported using stimulants, such as caffeine and energy drinks, which are known to be prevalent among this worker group (Jahnke and Kaipust, 2017). However, these stimulants are often accompanied by deleterious physiological effects. While studies have shown caffeine's efficacy in lab settings (Lorist and Tops, 2003) and effectiveness in other occupational domains (Horne and Reyner, 1996; Phillips et al., 2017), caffeine has considerable short-term (e.g., post-shift carryover effects, dehydration) and long-term (i.e., addiction, cardiovascular health) side effects (Kaur et al., 2020). They have deleterious health effects on occupational groups (Manchester et al., 2017; Higbee et al., 2020) and can be dangerous for ER workers, given the level and frequency of exertion and physiological stress they experience. Shift duration limits (Patterson et al., 2018), education programs (Sullivan et al., 2017), and sleep disorder screening (Barger et al., 2016) have been proposed as countermeasures.

The respondents in our study identified the first two as ideal countermeasures. However, they, as well as the literature, have noted the challenges associated with such proposals (Monday, 2000). Furthermore, such programs address sleep fatigue and cannot address fatigue that arises during the shift (Jeklin et al., 2020). Researchers in other domains have explored blue light exposure (Taillard et al., 2012) and alarm technology (Heinzmann et al., 2008). These methods are impractical in the dynamic and rugged physical environments in which ER workers perform their work and address limited populations of ER (e.g., drivers). The study responses identified a need for an effective countermeasure and that existing methods were suboptimal.

### 4.2 Interested but with caution

Several papers have delved into ethical considerations of using tDCS in clinical decision support systems, as well as for enhancement purposes (Lavazza, 2019; Davis and Smith, 2019; Feltman et al., 2020; Day et al., 2022; Auvichayapat and Auvichayapat, 2022). Among the ethical considerations raised in the literature are long-term benefits and risks, use in vulnerable populations, and the potential to change behavior and personality in unwanted or unforeseen ways.

Concerns raised for closed-loop architecture systems often discussed considerations about privacy, autonomy, and trust (Goering et al., 2021; Fairclough, 2014; Cabrera and Weber, 2023). While indeed some of our participants raised these concerns, several wanted to know more about the technology before they could have an opinion on it. A key concern was related to autonomy, the ability of users to keep some control over the system. Participants wanted to be part of the loop and have some decision on when to stimulate, rather than having the system do this to them without prior notification. Similar concerns have been discussed in the neuroethics literature regarding closed-loop neuromodulation systems (Tacca and Gilbert, 2023; Friedrich et al., 2018). For example, if a system can predict the onset of a seizure, should the patient be notified before stimulation is delivered to mitigate the seizure? Or should this happen without alerting the patient? Our participants preferred to be notified and then have the decision to on-demand stimulate. However, a few also mentioned that once they were more familiar with the system and trusted it, they would be comfortable with having the stimulation delivered without having “control” of this. It remains an empirical question if such an on-demand dosage of neuromodulation would be more efficient and safer than caffeine or other stimulants.

While the current neuroethics literature has hyper-focused on privacy concerns, this was not the most frequent concern, but over half of our participants did raise this as a concern. In particular, and in line with some of the current concerns highlighted in the literature around consumer neurotechnology, it is key to ensure that neural data recorded to inform the system is protected so that this information is not misused by insurance company policies to raise premiums or by employers for liability purposes. Midha et al. (2022), in a study looking at the perspectives of users who had experience tracking their mental workload, mentioned privacy as a key concern. While the participants in the said study feared the lack of protection from data mining, our participants cared more about this data being used by employers in the workplace to possibly evaluate them unfairly. Concerns about misuse of personal data in the workplace have been central in other emergent technology discussions, including genetics, which later led to the Genetic Information Non-discrimination Act (GINA; Kostiuk, 2012). While some scholars have suggested similar protection for neural data, it remains to see how governance of these types of data will take place in general and in particular in the workplace. While some participants raised safety considerations, in controlled protocol settings, tDCS has shown limited deleterious effects over repeated usage (Bikson et al., 2009), yet considering the on-demand nature of the system is an open question if there might be unanticipated health effects, which calls for a cautious approach as the system continues to be developed and deployed (Bikson et al., 2013; Riggall et al., 2015). Our results showed that some first responders questioned the kinds of side effects or future implications this brain stimulation would bring. In using tDCS to help with fatigue post-COVID-19, a study (Oliver-Mas et al., 2023) found that participants experienced minimal adverse effects and deemed it relatively safe, however, using it for fatigue in workplace safety is yet to be explored fully.

Finally, misuse and mistrust are another set of concerns that the literature on novel neurotechnologies has raised (Goering et al., 2021; Cabrera and Weber, 2023). While some recent work focused on trust at the intersection of how AI is used in the context of neurotechnologies (Yuste et al., 2017), our respondents were very much focused on how employers or other entities (such as insurance or legal systems) might misuse the data collected to harm the users. Participants also raised the concern of pushing a person past their natural limits as another example of misuse. This concern is in particular relevant in light of the vast literature around the use of neurostimulation technologies for enhancement (Hamilton et al., 2011; Lapenta et al., 2014; Cabrera et al., 2014; Willms and Virji-Babul, 2020), where proponents see the ability to push past our “natural” limits as a good thing, and thus suggest that we should embrace neurotechnologies that help with that. In the context of the workplace, for example, some scholars have written on the use of stimulants or AI to help workers stay attentive (The New York Times, 2015; Hosseini et al., 2023). However, while the case has been made for neurotechnologies for aiding soldiers during military applications, a pertinent question to answer is whether emergency response is considered a typical workplace or critical work within national security.

A related concern is mistrust. In a survey by Riggall et al. (2015), participants raised concerns about the inappropriate use by non-experts. Connected to misuse is the concern of the potential for forced use by employers (Cabrera and Weber, 2023). While these were not the main concerns among our respondents, the majority certainly voiced wanting to have the option to decide whether to use or not the system, in particular in light of the lack of rigorous evidence about how the benefits outweigh any potential risks.

### 4.3 Diverse views on acceptability

Considering the little awareness around neuromodulation technologies in general (Tyron et al., 2023) and the influence of contemporary science fiction and popular media, where neurostimulation is often portrayed as akin to mind control, it is key to understand the mediators shaping users' acceptance of these technologies. For example, Cabrera and Reiner (2015) found that while misunderstanding about tDCS has decreased as the technology matures, the public continues to have a cautionary and, at times, skeptical view about tDCS as a cognitive support tool, which is an important consideration for ER applications. While tDCS has demonstrated high efficacy and safety in managing fatigue in clinical populations, there has not been a specific discussion about the potential use of tDCS as a fatigue intervention in critical work domains such as ER. As such, the design and evaluation of interventions should be informed by socio-behavioral and neuroethics considerations with a user-centered approach. For several of our participants, the user-friendliness of the system was a key aspect to facilitate its use. Similar to previous research, our participants agree that if they knew more about the system, they would be more likely to try it. Moreover, given the already high demand type of workplace, the majority of participants mentioned that for the system to be uptake, it should not interfere with their work (e.g., get stuck with other equipment or gear) and that it would need to be resilient to the sometimes-extreme work conditions that some first respondent works endure (e.g., high heat conditions). Interestingly, several participants wanted a system that didn't make them look funny as they were afraid that that would affect their relationship with those they aimed to serve. Finally, several considered that if the system is effective in mitigating fatigue challenges, acceptance of the technology could be rapidly scaled by approval of public safety regulatory agencies, such as the National Fire Protection Agency.

### 4.4 Limitations

Several groups and reports have pointed to the necessity to anticipate the effects of implementing neurotechnology in applications beyond medical and wellness (OECD, 2019; Subgroup BRAIN, 2023; IEEE BRAIN, 2024), in this way, our study was valuable as it provided granular, real-world insights from potential users regarding the ethical concerns and acceptability of a neurotechnology-based system for first emergency responders. It is important to acknowledge that our results are only based on a small sample of ER workers, which may differ from the opinion of other samples. Furthermore, there is a wide heterogeneity of roles in ER workers, which certainly plays a role in their perspectives and concerns with such a system. Therefore, while the findings raise important considerations, much more research is needed to understand the nuances of neurotechnology in different workplace settings. It should also be noted that the type of neurotechnology under discussion is a very specific type of neurotechnology system aimed as a fatigue countermeasure, which means that some of our findings might differ from what is commonly seen in the literature concerning the ethics of consumer neurotechnology.

## 5 Conclusion

This study presented a novel empirical approach to understanding ethical concerns and acceptability surrounding a system currently being developed as a fatigue countermeasure. Our results relating to user control, privacy, and potential misuse align with concerns discussed for consumer neurotechnology. The results relating to safety and mistrust highlight important considerations that should be explored further. Continuous interdisciplinary dialogue on the ethical, legal, social, and cultural implications of the use of neurotechnology to support user cognition and health is critical, as shown by the efforts led by IEEE BRAIN and their proposed framework around different applications of neurotechnology (IEEE BRAIN, 2024).

## Data Availability

The de-identified data supporting the conclusions of this article will be made available by the authors, without undue reservation.
